# Perceived seriousness mediates the influence of cervical cancer knowledge on screening practices among female university students in Ghana

**DOI:** 10.1186/s12905-019-0842-y

**Published:** 2019-11-19

**Authors:** Francis Mensah Annan, Kwaku Oppong Asante, Nuworza Kugbey

**Affiliations:** 1 Department of Psychology, University of Ghana, Legon, Accra, Ghana; 2 Department of Psychology, University of the Free State, Bloemfontein, South Africa; 3 Department of Family and Community Health, University of Health and Allied Sciences, Hohoe Campus, Hohoe, Volta Region Ghana

**Keywords:** Cervical cancer, Female university students, Perceived risks screening behaviour, Susceptibility

## Abstract

**Background:**

Cervical cancer knowledge and awareness, as well as an individual’s perceptions about cervical cancer have been shown to significantly influence the screening practices of female students. Despite these studies, the mechanisms linking cervical cancer knowledge to screening practices among female students remain unexplored in the literature. Thus, this study examined the direct and indirect influences of cervical cancer knowledge on screening practices through perceptions about cervical cancer as informed by the health belief model.

**Methods:**

A cross-sectional survey design with a purposive sample of 200 female students were used in the study. Standardized questionnaires were used to measure cervical cancer knowledge, perceived susceptibility, perceived seriousness, perceived benefits, perceived barriers and cervical cancer screening behaviours. The Pearson product-moment correlation co-efficient and mediation analyses were used to analyse the data.

**Results:**

Our findings showed that cervical cancer knowledge, perceived susceptibility, perceived seriousness and perceived benefits were significant and positively correlated with increased screening behaviours. However, only perceived seriousness significantly mediated the relationship between cervical cancer knowledge and screening behaviour. Cervical cancer knowledge remained a significant direct predictor of screening behaviour in all the models.

**Conclusion:**

These findings underscore the need for increased awareness with emphasis on the seriousness of cervical cancer among female university students as it plays a key role in influencing their screening behaviours.

## Background

Cervical cancer is the fourth most frequent cancer in women, with an estimated 570,000 new cases in 2018 representing 6.6% of all female cancers [1]. In Ghana, cervical cancer is one of the most diagnosed cancers among women [2, 3]. Cervical cancer results from irrepressible growth and spread of atypical cells in the cervix of the female reproductive organ. It has become a major public health concern due it’s associated high mortality rate [3, 4]. Human papilloma virus (HPV) is responsible for almost all cervical cancers, and it is linked to increased number of sexual partners and early sexual debut [5, 6]. Additionally, smoking, poor diet, excessive use of oral contraceptive pills and sexually transmitted infections (STIs) have been identified as risk factors for cervical cancer among women [7, 8].

The majority of the risk factors associated with cervical cancer are modifiable, an indication that lifestyle changes could be helpful in preventing the prevalence of cervical cancer among females [9, 10]. One of the effective ways of preventing cervical cancer is early screening and treatment [1]. However, several factors have been identified to influence cervical cancer screening practices among females [3, 4, 11]. Knowledge about cervical cancer is one of the key determinants of cervical cancer screening practices among females as evidenced in the cervical cancer literature [12–14]. It has been reported that females with high knowledge and increased awareness about cervical cancer are more likely to engage in cervical cancer screening practices [11, 15]. These studies, thus, imply that knowledge and awareness about cervical cancer are important in screening practices among females.

Besides awareness and knowledge of cervical cancer, individual’s perception about cervical cancer as informed by the health belief model have been shown to have significant influence on screening practices of females [16, 17]. For instance, perceived benefits, perceived susceptibility, perceived severity/seriousness, and perceived barriers have been documented in the literature to be associated with cervical cancer screening practices [17, 18]. Evidence also suggests that knowledge about cervical cancer also influence individual’s perception about the disease [19]. It is clear in the literature that cervical cancer knowledge and perceptions as informed by the health belief model are significant predictors of cervical cancer screening practices [20, 21].

Studies conducted among female students in institutions of higher education in Ghana have revealed that lack of knowledge on specific risk factors, had relatively fair perception of cervical cancer and showed poor cervical cancer screening behaviour [11, 12]. It has also been shown that the lack of belief that: cervical screening diagnoses cancer; pap test is painful; and that cervical cancer screening will take away one’s virginity were barriers that influence cervical cancer screening [22, 23]. Despite these studies, the mechanisms linking cervical cancer knowledge to screening practices among female students remain unexplored in the literature. Thus, this study examined both the direct and indirect influence of cervical cancer knowledge on screening practices through perceptions about cervical cancer as informed by the health belief model. It is believed that factual knowledge and awareness about cervical cancer may influence beliefs and perceptions of females about cervical cancer which may in turn affect their screening practices. The result from this study would shed light on the significant mechanisms through which cervical cancer knowledge influences screening practices among female university students in Ghana as the majority of the previous studies were descriptive in nature without examining the possible complex interrelationships among the factors that could influence the screening behaviours.

## Methods

### Research design and participants

The study used a cross-sectional survey design. This design was used because it entails surveys or other pre-structured means that helped to obtain a common dataset on some pre-selected variables. Research participants were selected using a purposive sampling approach where the researchers sampled participants based on their ability to provide rich information that would help achieve the objectives of the study. Snedecor and Cochran’s [24] formula for sample size calculation was used; $n=\frac{{\left({Z}_{\alpha /2}\right)}^2p\left(1-p\right)}{d^{2.}}$

Where:

$$ n=\mathrm{sample}\ \mathrm{size}\ \mathrm{required} $$

$$ {\left({Z}_{\frac{\alpha }{2}}\right)}^2=\mathrm{reliability}\ \mathrm{co}-\mathrm{efficient} $$

$$ p=\mathrm{proportion}\ \mathrm{of}\ \mathrm{study}\ \mathrm{population} $$

$$ {d}^2=\mathrm{margin}\ \mathrm{of}\ \mathrm{error} $$

$$ {Z}_{\alpha /2}=1.96\  at\ 95\% confidence\ level $$

*p*= 8.0% or 0.08,prevalence of cervical cancer screening beahaviour reported by Binka, Nyarko and Doku [11].

$$ {d}^2=5\%\mathrm{or}\ 0.05 $$

Therefore, $$ \mathrm{n}=\frac{\left(1.96\ x\ 1.96\right)\ x\ 0.08\left(1-0.08\right)}{0.05\ x\ 0.05}=113 $$

Thus, a minimum of 113 participants was required. We sampled a total of 200 female undergraduate university students in a public university in Ghana which we found to be adequate for the study.

### Procedure

Ethical clearance to conduct the study was obtained from the Research and Ethics Committee of the Department Psychology, University of Ghana. Those who participated in the study met the following 2 inclusion criteria which are be a female registered student; and should not be less than 16 years old. During the period of collecting data, the first author approached females in various locations within the university and invited them to participate in the study. All the key ethical principles of informed consent, voluntary participation, anonymity and confidentiality were adhered to during the data collection process. Administration and completion of questionnaire by the participant was approximately 30 min. Students were neither reimbursed nor induced for their participation. Data was collected over a period of 3 weeks.

### Measures

In this study, the outcome variable was cervical cancer screening behaviour with cervical cancer knowledge as the independent variable. Additionally, four (4) mediating variables: perceived susceptibility to cervical cancer, perceived seriousness of cervical cancer, perceived barriers and perceived benefits cervical cancer screening were included in the study.

#### Cervical Cancer screening behaviour

Cervical cancer screening behaviour was assessed using 4 items from previous research [11]. These items were used to assess female students’ screening behaviour. Some sample items included “Have you ever screened for cervical cancer in the last 2 years?” and “Do you have any intention of getting screen for cervical cancer in the future?” Responses to the items were in a form of yes = 1 and no = 0. Participants responses were scored such that higher scores indicated higher screening behaviour.

#### Perceived susceptibility to cervical Cancer

Perceived susceptibility to cervical cancer was assessed using 8 items from previous research [17, 19]. These items were used to assess female students’ perceived susceptibility to getting cervical cancer. Some sample items included “Women with multiple sexual partners are more prone to cervical cancer disease” and “Older women are more at risk of cervical cancer than younger women”. Scale responses ranged from 1 (strongly disagree) to 5 (strongly agree). Participants responses were scored such that higher scores indicated greater perceived susceptibility to getting cervical cancer.

#### Perceived seriousness of cervical Cancer

A seven-item measure adapted from previous studies [17, 19] were used to assess female students’ perceived seriousness of cervical cancer. The response options are rated on a 5-point Likert-type scale ranging from 1 (*strongly disagree*) to 5 (*strongly agree*). Sample of the items included “Having cervical cancer will make a woman’s life difficult”, and “Cervical cancer reduces life span of women”. Higher scores on this measure indicate greater perceived seriousness of cervical cancer.

#### Perceived benefits of cervical Cancer screening

A six-item measure adapted from previous studies [17, 19] were used to assess female students’ perceived benefits of cervical cancer screening. The response options are rated on a 5-point Likert-type scale ranging from 1 (*strongly disagree*) to 5 (*strongly agree*). Higher scores on this measure indicate higher perceived benefits of cervical cancer screening. Sample items on this subscale include “It is important for a woman to have cervical cancer screening to know if she is healthy” and “Cervical cancer screening brings about long, enjoyable life for the woman”.

#### Perceived barriers to cervical Cancer screening

Perceived barriers to cervical cancer screening was assessed using 15 items selected from previous research [18, 25]. Some of the items on this subscale include “It is too embarrassing to undertake cervical cancer screening” and “My partner will not want me to undertake cervical cancer screening.” The response options were rated on a 5-point Likert-type scale ranging from 1 (*strongly disagree*) to 5 (*strongly agree*). Higher scores on this measure indicate greater perceived barriers to cervical cancer screening.

#### Cervical Cancer knowledge

Cervical cancer knowledge was assessed using 15 items selected from previous research [17, 19]. These 15 items are in a form of multiple-choice questions, with only one correct response for each question. A sample item is “The virus associated with cervical cancer is transmitted by: a. Sexual intercourse, b. Maternal-fetal transmission, c. Blood transfusions, d. Inanimate objects, e. I don’t know” A participant receives a score of 1 for a correct response and 0 for an incorrect response. Thus, scores can range from 0 to 15 with higher scores indicating greater knowledge on cervical cancer

### Data analyses

The Statistical Package for the Social Sciences (SPSS) version 24.00 was used to analyze the data. Pearson’s correlation analysis was used to test the relationships among the study variables. Mediation analysis was done using PROCESS Macro [26]. The PROCESS Macro is an add-on in SPSS which allows researchers to examine both direct and indirect effects of independent variables on a dependent variable(s) using the ordinary least squares (OLS) regression method. This method has been considered as a more robust statistical technique than traditional mediation analyses as it does not make any assumptions about the normality of the data [26]. In the mediation analysis, four separate models were tested with each of the four constructs of the health belief model used in separate mediation analysis (perceived susceptibility, perceived seriousness, perceived benefits and perceived barriers). Separate models were used for the mediation as the mediators are correlated significantly. To test the indirect effects of the cervical cancer knowledge on the screening practices among the participants, 5000 bootstrap re-samples were used, and 95% confidence intervals were used to determine the significance of the effects. If the 95% confidence intervals included zero (0), then the indirect effects of the cervical cancer knowledge on the screening practices is not significant. On the other hand, if the 95% confidence intervals were entirely above or below zero (0) then a significant indirect effect is assumed. All analyses were two-tailed, and a *p* ≤ 0.05 was considered statistically significant.

## Results

### Demographic profile of the participants

The mean age of the participants was 20.4 years [*Standard Deviation (SD)*] = 1.96) with the age range of 17–26 years. Over a third of the participants were first (38.0%) and second (32.5%) year students. Approximately 92% of the participants. The results further showed that 95% of the participants religious affiliation was Christianity. Approximately 80% of the participants reported to have lived in an urban area when growing up. Table 1 below shows the demographic characteristics of the sample.

**Table 1:** Socio-demographic characteristics of participants (*N* = 200)

Variable	Frequency	Percentage (%)
Marital Status		
Single	156	78.0
In relationship	42	21.0
Married	2	1.0
Area of residence		
Urban	160	80.0
Semi -urban	34	17.0
Rural setting	6	3.0
Year of study		
First year	76	38.0
Second year	65	32.5
Third year	35	17.5
Fourth year	24	12.0
Family background		
Wealthy (within the top 25%)	26	13.0
Quite well off (within the 50 to 75% range)	157	78.5
Not well off (within the 25 to 50% range)	17	8.5
Religion		
Christian	189	94.5
Muslim	9	4.5
Other	2	1.0
Working status		
Working	10	5.0
Non-working	190	95.0

### Descriptive statistics of the study variables

The descriptive statistics of the study variables are presented in Table 2. Results from Table 2 show that all the scales used in measuring the study variables demonstrated good reliability values in exception of perceived barriers which was a little below .70.

**Table 2:** Summary of descriptive statistics of the study variables

Variables	Total items	Range	Mean	SD	Reliability
Knowledge	15	0–15	4.90	3.05	–
Susceptibility	8	8–40	25.43	3.85	.84^a^
Seriousness	7	7–35	25.95	3.38	.81^a^
Benefits	6	6–30	21.61	2.71	.72^a^
Barriers	15	15–75	47.12	6.81	.68^a^
Screening	4	0–4	1.15	0.76	.74^b^

^a^ = Cronbach alpha, ^b^ = Kuder-Richardson’ (KR-20) coefficient, SD = Standard Deviation

### Bivariate relationships among the study variables

Findings from the correlation analyses showed that increased cervical cancer knowledge is significantly related with increased perceived susceptibility (*r* = .37, *p* < .001), perceived seriousness (*r* = .31, *p* < .001), perceived benefits (*r* = .16, *p* < .05) and cervical cancer screening behaviours (*r* = .26, *p* < .001). It was also found that perceived susceptibility (*r* = .19, *p* < .01), perceived seriousness (*r* = .22, *p* < .01), perceived benefits (*r* = .15, *p* < .05) were significantly and positively correlated with cervical cancer screening behaviours among female students. However, cervical cancer knowledge (*r* = .05, *p* > .05) and screening behaviours (*r* = −.02, *p* > .05) were not significantly correlated with perceived barriers to cervical cancer screening. These results are summarized in Table 3 below.

**Table 3:** Correlation matrix of the relationships among the study variables

Variables	1	2	3	4	5
1. Cervical cancer knowledge	1				
2. Perceived susceptibility	.37***	1			
3. Perceived seriousness	.31***	.34***	1		
4. Perceived benefits	.16*	.19**	.39***	1	
5. Perceived barriers	.05	.24**	.01	.11	1
6. Screening Behaviour	.26***	.19**	.22**	.15*	−.02

^*^p < .05; ^**^p < .01; ^***^p < .001

### The direct and indirect effects of cervical cancer knowledge on screening behaviours

#### The mediating effect of perceived susceptibility on the link between knowledge and screening practices

**Fig. 1:**
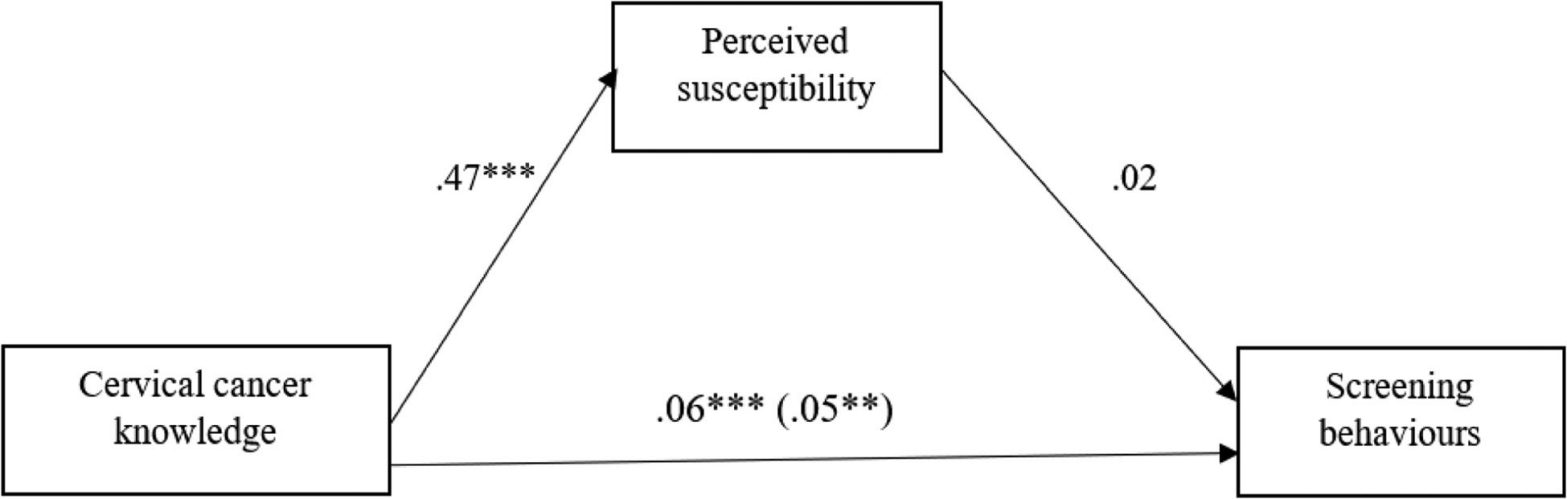
Summary mediating effect of perceived susceptibility on the link between cervical cancer knowledge and screening practices

As can be observed from Fig. 1, increased cervical cancer knowledge predicted increased perceived susceptibility to cervical cancer (*b* = .47, *p* < .005). However, perceived susceptibility to cervical cancer did not significantly predict screening practices among the participants (*b* = .02, *p* > .05). The indirect effect of cervical cancer knowledge on screening behaviour through perceived susceptibility was not significant (*b* = .0105) as the confidence intervals included zero (−.0028 to .0243). There was however, evidence of direct significant influence on cervical cancer knowledge on screening behaviours (*b* = .05, *p* < .01).

#### The mediating effect of perceived seriousness on the link between knowledge and screening behaviours

**Fig. 2:**
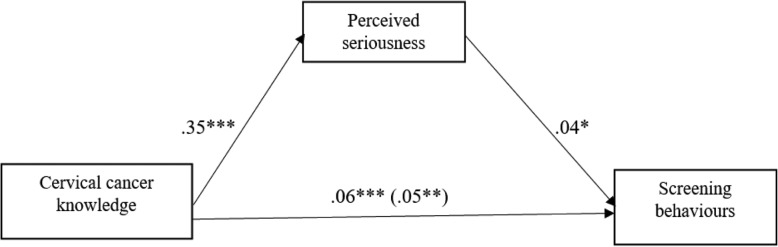
Summary mediating effect of perceived seriousness on the link between cervical cancer knowledge and screening behaviours

As can be seen in Fig. 2 below, increased cervical cancer knowledge predicted increased perceived seriousness of cervical cancer (*b* = .35, *p* < .001). Increased perceived seriousness significantly predicted increased cervical cancer screening behaviours (*b* = .04, *p* < .05). The Indirect effect of cervical cancer knowledge on screening behaviour through perceived seriousness was significant (*b* = .0123) as the confidence intervals were entirely above zero (.0012 to .0270). However, there was evidence for the direct predictive influence of cervical cancer knowledge on screening behaviours among the participants (*b* = .05, *p* < .05).

#### The mediating effect of perceived benefits on the link between cervical cancer knowledge and screening behaviours

**Fig. 3:**
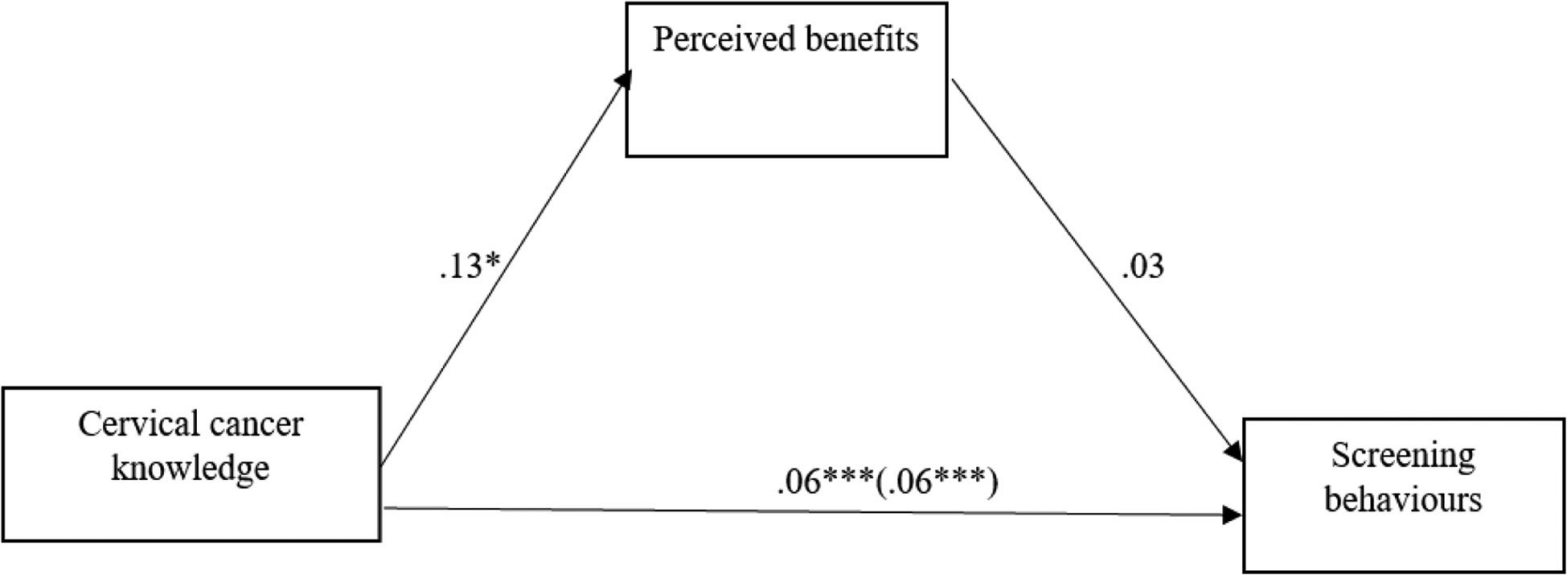
Summary of the mediating effect of perceived benefits on the link between cervical cancer knowledge and screening behaviours

Figure 3 below shows that increased cervical cancer knowledge predicted increased perceived benefits of screening behaviours (*b* = .13, *p* < .05). However, perceived benefits of cervical cancer screening did not significantly predict actual screening behaviours (*b* = .03, *p* > .05). There was also no significant indirect effect of cervical cancer knowledge on screening behaviours through perceived benefits (*b* = .0045), as the confidence intervals included zero (−.0010 to .0136). Increased cervical cancer knowledge had a significant direct effect on screening behaviours (*b* = .06, *p* < .001).

#### The mediating effect of perceived barriers on the link between cervical cancer knowledge and screening behaviours

**Fig. 4:**
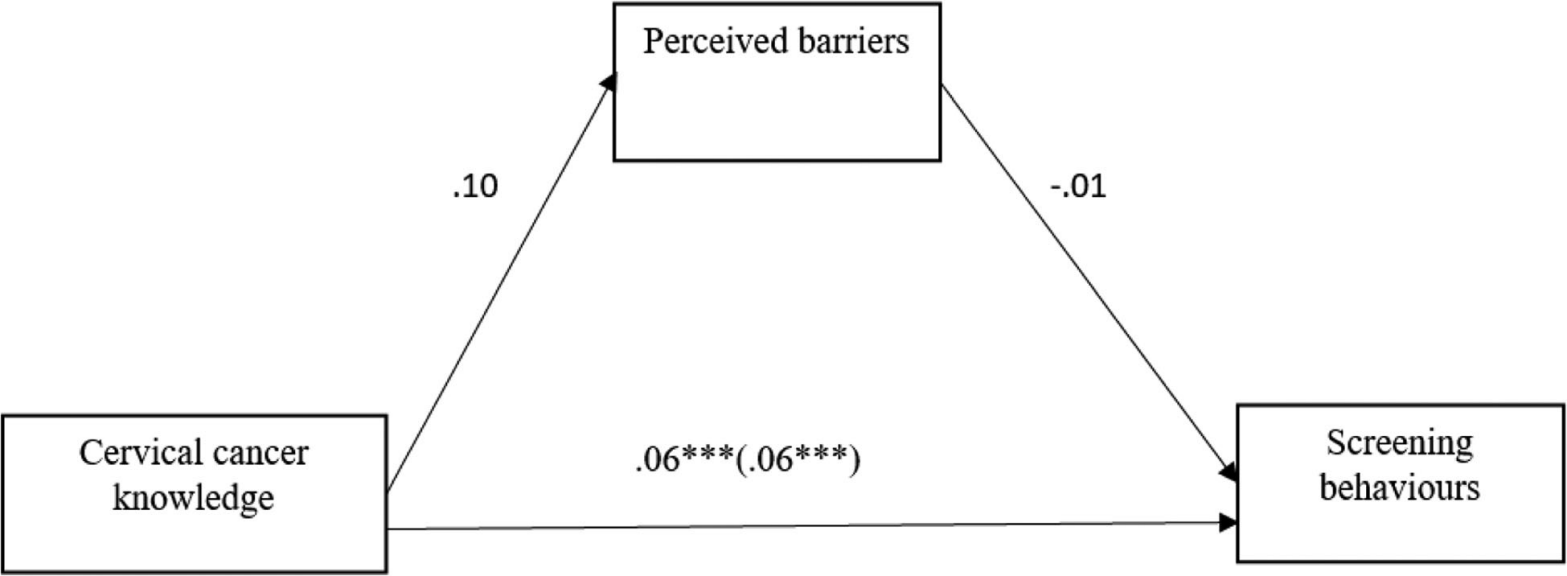
Summary of the mediating effect of perceived barriers on the link between cervical cancer knowledge and screening behaviours

As can be seen in Fig. 4 below, cervical cancer knowledge did not significantly predict perceived barriers to cervical cancer screening (*b* = .10, *p* > .05). Perceived barriers to cervical cancer screening did not significantly predict cervical cancer screening behaviours (*b* = −.01, *p* > .05). The Indirect effect of cervical cancer knowledge on screening through perceived barriers was not significant (*b* = −.0003) as the confidence intervals included zero (−.0048 to .0024). However, there was evidence for the direct predictive influence of cervical cancer knowledge on screening behaviours among the participants (*b* = .06, *p* < .001).

## Discussion

This study examined the direct and indirect effects of cervical cancer knowledge on screening practices through perceptions (health belief model) among female university students in Ghana. Our results showed that cervical cancer knowledge, perceived susceptibility, perceived seriousness and perceived benefits were significant and positively correlated with increased cervical cancer screening behaviours. In the mediation analyses, only perceived seriousness significantly mediated the relationship between cervical cancer knowledge and screening behaviours. However, cervical cancer knowledge remained a significant direct predictor of screening behaviours in all the models involving the mediators.

Findings showed that cervical cancer knowledge was significant and positively associated with screening practices among female university students. It was further observed that cervical cancer knowledge remained a significant predictor of screening practices in all the mediation models. This finding suggests that increasing cervical cancer knowledge and awareness influence female students’ chances of getting tested for cervical cancer and engaging in related behaviours. Increasing cervical cancer knowledge and its relationship with screening behaviours as found in this study are not surprising as the literature is replete with evidence of this significant positive relationship [7, 11–15]. This finding emphasizes the importance of factual knowledge on cervical cancer as this is likely to promote effective screening practices among females of reproductive age.

The health belief model constructs such as perceived susceptibility, perceived seriousness and perceived benefits were significant and positively correlated with increased cervical cancer screening behaviours in this study. Evidence abound in health promotion and education literature on the impact of health belief constructs on screening behaviours among different populations. For examples, studies have found perceived susceptibility, perceived seriousness and perceived benefits of cervical cancer screening to be key determinants of cervical cancer screening practices [13, 18, 20, 21].

Mediational results showed that only perceived seriousness significantly mediated the relationship between cervical cancer knowledge and screening behaviours. It was observed that increased cervical knowledge was associated with increased perceived seriousness of cervical cancer which in turn results in increased cervical cancer screening practices. Cervical knowledge significantly predicted perceived susceptibility, perceived seriousness and perceived benefits of cervical cancer screening among female university students. However, only perceived seriousness significantly predicted increased cervical cancer screening practices. The significant mediation effect by perceived seriousness of cervical cancer on the link between cervical cancer knowledge and screening practices has been reported by some researchers [19, 21]. This finding is however, inconsistent with previous works which found most of the health belief model constructs to be significant mediators of the link between cervical cancer knowledge and screening practices [20] The inconsistency may be due to the composition of the sample size as the current study was among female university students as against a community-based sample.

The findings from this study have some implications for health promotion and education activities as well as cervical cancer research. Health promotion and education activities among female university students should be targeted at providing students with factual information about cervical and its associated risk factors, signs and effects. This is likely to help female students change their beliefs and perceptions as well as their cervical cancer screening practices. Future research in the area of cervical cancer should examine these health beliefs constructs among community-based samples to determine all the possible factors that influence cervical cancer screening.

Like any other studies, this study is not without limitations. The use of a cross-sectional study and selecting only four of the health belief model constructs serve as limitations to the findings from this study. Also, the relatively small sample size may also impact on the generalisability of the findings. and the use of a cross-sectional design as no causal inferences could be drawn between the variables used in this study. A more larger representative sample of female students from other institutions of higher learning could increase the generalizability of the results. There was also the potential of responses to be affected by social desirability as the study relied on self-report measures. Despite these shortfalls, this is one of the few studies within the African context to have used key constructs in the health belief model in explaining cervical cancer screening behaviour.

## Conclusion

This study was conducted to examine the direct and indirect effect of cervical cancer knowledge on screening practices through perceptions about cervical cancer as informed by the health belief model. Our result showed only perceived seriousness significantly mediated the relationship between cervical cancer knowledge and screening behaviours. However, cervical cancer knowledge remained a significant direct predictor of screening behaviours in all the models involving the mediators. Thus, cervical cancer knowledge plays an important role in influencing the perceptions regarding cervical cancer and screening behaviours among students. Therefore, health intervention should take into consideration awareness creation and provision of accurate information to achieve optimum screening behaviours to reduce the incidence of cervical cancer and associated physical, economic and psychosocial problems.

## Data Availability

All data generated during and/or analyzed during the study are available from the corresponding author on reasonable request.

## Abbreviations

HPV: Human papilloma virus
OLS: Ordinary Least Squares
SD: Standard Deviation
SPSS: Statistical Package for the Social Sciences
STIs: Sexually Transmitted Infections
